# A perpetual source of DNA or something really different: ethical issues in the creation of cell lines for African genomics research

**DOI:** 10.1186/1472-6939-15-60

**Published:** 2014-08-07

**Authors:** Jantina de Vries, Akin Abayomi, James Brandful, Katherine Littler, Ebony Madden, Patricia Marshall, Odile Ouwe Missi Oukem-Boyer, Janet Seeley

**Affiliations:** 1 Department of Medicine, University of Cape Town, Office J52-16, UCT Centre for Clinical Research, Old Main Building, Groote Schuur Hospital, Observatory, 7925 Cape Town, South Africa; 2 NSB-H3A biobank and the National Health Laboratory Services of South Africa, Stellenbosch University, Faculty of Medicine and Health Sciences, Private Bag X1, Matieland, 7602 Stellenbosch, South Africa; 3 Noguchi Memorial Institute for Medical Research, University of Ghana, Legon, PO Box LG 581, Ghana; 4 Wellcome Trust, Gibbs Building, 215 Euston Road, London NW1 2BE, UK; 5 Division of Genomic Medicine, National Human Genome Research Institute, Building KEYSTN, Room 3130, 530 Davis Dr, MSC K3-02, Durham NC 27713-K3-02, England; 6 Department of Bioethics, School of Medicine, Case Western Reserve University, 10900 Euclid Avenue, Cleveland, Ohio 44106-4976, USA; 7 CERMES, 634 Bd de la Nation, PO Box 10887, YN034 Niamey, Niger; 8 Cameroun Bioethics Initiative (CAMBIN), The Ark, Mendong, PO Box 31489, Yaoundé, Cameroon; 9 MRC/UVRI Uganda Research Unit on AIDS, P.O. Box 49, Entebbe, Uganda

**Keywords:** Cell lines, H3Africa, Africa, Ethics, Consent, Sample ownership, Immortalisation

## Abstract

**Background:**

The rise of genomic studies in Africa – not least due to projects funded under H3Africa – is associated with the development of a small number of biorepositories across Africa. For the ultimate success of these biorepositories, the creation of cell lines including those from selected H3Africa samples would be beneficial. In this paper, we map ethical challenges in the creation of cell lines.

**Discussion:**

The first challenge we identified relates to the moral status of cells living in culture. There is no doubt that cells in culture are alive, and the question is how this characteristic is relevant to ethical decision-making. The second challenge relates to the fact that cells in culture are a source of cell products and mitochondrial DNA. In combination with other technologies, cells in culture could also be used to grow human tissue. Whilst on the one hand, this feature increases the potential utility of the sample and promotes science, on the other it also enables further scientific work that may not have been specifically consented to or approved. The third challenge relates to ownership over samples, particularly in cases where cell lines are created by a biobank, and in a different country than where samples were collected. Relevant questions here concern the export of samples, approval of secondary use and the acceptability of commercialisation. A fourth challenge relates to perceptions of blood and bodily integrity, which may be particularly relevant for African research participants from certain cultures or backgrounds. Finally, we discuss challenges around informed consent and ethical review.

**Summary:**

In this paper, we sought to map the myriad of ethical challenges that need to be considered prior to making cell line creation a reality in the H3Africa project. Considering the relative novelty of this practice in Africa, such challenges will need to be considered, discussed and potentially be resolved before cell line creation in Africa becomes financially feasible and sustainable. We suggest that discussions need to be undertaken between stakeholders internationally, considering the international character of the H3Africa project. We also map out avenues for empirical research.

## Background

Genomic research in Africa has received significant support under a funding programme called ‘H3Africa’, where H3 stands for Humans, Heredity and Health. H3Africa ( http://www.h3africa.org) is a joint funding initiative by the Wellcome Trust in the United Kingdom and the National Human Institutes of Health (NIH) in the United States, with support from the African Society of Human Genetics. H3Africa supports individual research projects, a bioinformatics network (H3A BioNet) and a small number of African biorepositories to store (aliquots of) samples.^a^

Central to the establishment of biorepositories on the African continent (and elsewhere) is the creation of cell lines as a value-added service. A cell line is made by taking a human body cell such as a blood cell or skin cell and changing some of its’ genetics in order to make the cell reproduce indefinitely outside of the body. Once established, the cells in the cell line will keep on reproducing in a flask as long as they are taken care of – in other words, as long as they are fed and refreshed, and have oxygen to breathe. Because the cells in culture originate from a single cell, they are genetically uniform and share the genetic makeup of the person from whom the cell was obtained.

At the most basic level, the immortalisation of cells is considered as the optimal way to preserve DNA outside the human body. Once cell lines are created, they form an inexhaustible resource for DNA and, of course, other cell products too. A second advantage to the creation of cell lines, therefore, lies in that they may enable functional genomic work to be conducted. Whereas a sample collected at one moment in time may allow for only very limited functional work (e.g. RNA expression studies), a cell line allows researchers to study cellular responses to different stimuli that emulate infection or disease. In this way, it allows researchers to identify not only genetic factors involved in disease causation or prevention, but also to study the actual effect of those factors. A third advantage is that the collection of new samples is not necessary and thus may minimise the burden and cost of patient recruitment. The preservation of cells in culture allows for the application of the latest scientific techniques on these samples. An example of such a technology in development is single-cell genomics, which allows for detailed genomic and transcriptomic studies on the basis of a single cell [1,2].

However, the creation of cell lines also raises ethical issues, not all of which are thoroughly understood. In the African context, the creation of cell lines is relatively novel. In this paper, we will map the ethical issues around the creation of cell lines and discuss those that need to be addressed before the development of African sample repositories, as discussed by the H3Africa Working Group on Ethics.

### The historical creation of cell lines from African samples

The creation of cell lines became technologically feasible in the 1950s, when the first cell line that multiplied in vitro was generated using the cells of a cancer patient called Henrietta Lacks, also known as HeLa [3]. Since then, cell biology has found other ways of generating cell lines, including the reprogramming of adult body cells into stem cells [4,5]. Induced Pluripotent Stem Cell (IPSC) technology allows for body cells to be transformed into stem cells which can be induced to develop into cells of specialised body tissues, for instance neurones or fat cells through a few intermediary steps. Theoretically, cells derived from cell lines could also be used for these purposes. This technology raises important ethical issues that need to be considered in the context of cell line immortalisation.

Researchers have created cell lines from human specimens for over five decades, and have also used this technology to create cell lines from African samples – many of which are currently preserved by the Coriell Institute in the USA. Specific examples are cell lines created for the Biaka pygmy population from the Central African Republic and the Mbuti pygmy population from the Democratic Republic of Congo.^b^ Many of the earlier samples were not collected purposively for the creation of cell lines. More recent research, however, has witnessed the collection of African human samples specifically for cell line creation. This is the case for the 90 HapMap cell lines derived from samples obtained from Yoruba people living in Ibadan, Nigeria [6], and for the samples included in the 1000 Genomes study [7]. The latter has generated cell lines for Luhya people from Kenya, Mende people from Sierra Leone and Esan people from Nigeria. Cell lines are also available for samples collected for 100 people from The Gambia with undisclosed ethnicity.^c^ Many more cell lines may have been developed from samples obtained from African people, but these collections are thought to be scattered across laboratories and universities across the globe, and no comprehensive record exists. In the context of H3Africa studies, the expectation is that it would ultimately be beneficial to create cell lines for (selected) samples so that samples will not be limited for future research and additional resources will not be needed for renewed sample collection but this is not currently economically and technologically feasible.

## Discussion

### Are cell lines really different?

A key question relevant to a discussion of the ethical issues relating to the creation of cell lines is whether this practice distinguishes itself from other forms of sample manipulation in a way that is morally relevant. In other words, are there aspects of the creation of cell lines that raise ethical concerns in and of themselves?

On one extreme, the creation of cell lines could be considered as a way of preserving genetic material, and of ensuring access to this material in the future. Proponents of this view argue that the technique minimises harm to participants by reducing the need for additional samples, whilst maximising benefit by enabling the development of a more comprehensive picture of disease, which will ultimately be of benefit to patients. From this perspective, the creation of cell lines is not morally different from the collection of a finite genetic sample.

An opposing view could be that cells in culture are living: they breathe oxygen, consume nutrients, multiply and can die. If they have the ability to die, then they logically need to be considered alive as well. This is significantly different from other genomic material collection which required the collection of a finite genomic sample. When considered in this way, the creation of cell lines touches on concepts that were previously associated with, for example, deities or superheroes – immortality and the ability to live and multiply outside of the body.

The latter observation – that cells in culture are alive – may be morally significant. Obviously, cell lines in a flask are not experiencing life in the same way that a living human does, and they should therefore not be treated the same as the conscious, living persons. They share with the humans from whom they came, all of the genetic material as well as the ability to live *in vitro* under artificially engineered circumstances. They have the ability to provide information on the physiological body of the person who donated them, but do not seem to have the same ability to provide information on the other aspects that make up a person – for instance, their emotional state, thoughts and lived experiences. That cells outside of the body do have meaning may be clear from a comparison with cells in semen [8]. However, to what degree these samples still ‘pertain to’ the person, or whether the sample donors retain ‘interests in’ the cell lines, is not currently clear and needs further discussion and investigation [9]. A complicating factor is that (collections of) cell lines may be used to provide information not only on the person, but on the population group that they form part of – a fear that is prevalent in the context of genomic research [10].

### Cells in culture: a source of cell products

A second way in which cell lines are different from finite genomic samples is that they offer a source of cell products other than just nuclear DNA. Cell lines offer access not only to mitochondrial DNA, but also to transcription RNA, expressed proteins and other cell products. This is morally relevant most importantly because it significantly increases the usefulness of the donated sample. For instance, rather than just examining the ‘genetic blueprint’, which is static, cell lines could be used to study (genetic) responses to environmental stimuli, including infection with disease-causing pathogens or therapeutic drugs. This means that rather than just focusing on an individuals’ genetic makeup and analysing the influence of genetic variants on disease, the creation of cell lines allows a researcher to study disease responses at the cellular level over time, without necessarily returning to the research participant for further samples or information, and without having to subject them to potentially harmful experiments. The expectation is that using cell lines will enable researchers to discover more about disease in a shorter timeframe – which would ultimately be beneficial for patients. Where projects focus on diseases prevalent in Africa, the expectation would be that the benefits from knowledge gained would accrue to African patients and the wider population, but it may be important to include this as a specific requirement in access decisions. If this expectation is true, then the creation of cell lines would have moral significance in ultimately promoting the wellbeing of African patients.

Access to mitochondrial DNA may also be relevant for consent as it provides information about group origin, but this may not specifically have been highlighted in the consent forms. A concern could be that genetic accounts of ancestry may be in conflict with traditional narratives – which was apparently the case for the Havasupai in the United States [11,12]. In that project, genetic material collected for diabetes-related studies from a group of Native Americans called the Havasupai was used to study the group’s origin. This was found to be offensive to the Havasupai and resulted in the destruction of all genetic samples held in storage. However, studies of ancestry do not need to include mitochondrial DNA, but could equally well be done on genomic material from the nucleus. Y-chromosomal material – which has the same potential as mitochondrial DNA to inform on ancestry – is routinely genotyped or sequenced in genomic studies and this has not raised significant concern to date. This aspect, therefore, is not unique to cell lines; there are similar challenges for research where only a finite DNA sample is collected.

### Using cells to grow other tissues

Cells grown in culture can also be used to grow other tissues, which is not possible if a finite sample is not converted to cell lines. Using novel techniques such as those developed for the creation IPSCs [4,5], researchers in the field of regenerative medicine could theoretically induce the cells in culture to transform into stem cells, and then subsequently get these to develop into cells of a different type. In other words, using IPSC techniques, researchers could theoretically manipulate white blood cells grown in culture to transform into muscular or neurological cells, for instance. This would be highly advantageous for science: for example, in a study of cardiovascular disease, one could grow cardiomuscular cells with the same genetic makeup as the cells of the patients recruited into the study. This could allow researchers to develop a much better understanding of disease processes, without having to go through complicated and sometimes painful procedures to extract patient-specific tissues from their bodies. What this means is that cell lines take on value and possibilities for application that may transcend the value and use of the original human body or person [13].

The use of these technologies generates a number of ethical concerns. One challenge relates to informed consent – whether the patient indeed agreed to allowing such manipulations to happen on the samples. Another challenge relates to immoral or illegal applications of these methods, for instance to support the creation of artificial gametes. Although this is currently illegal in most jurisdictions across the world, it is difficult to monitor how researchers use samples in the privacy of their laboratories.

### Ownership over cell lines

In the context of the H3Africa programme, the aim is to create biorepositories across a number of countries in Africa. The vision is that samples are shipped regionally, and that cell lines are created, maintained and distributed from these regional African centres to researchers elsewhere in the world. These biorepositories would be within African institutions and access decisions would be made by an oversight committee that is primarily composed of people residing in Africa. The intention to create African biorepositories seems to be a partial response to concerns about sample export and exploitation which have been raised in the context of genomic research [14-16]. The creation of regional African repositories, however, does not eliminate the need for sample export and concerns relating to ownership and sovereignty may be as relevant when samples are shipped from one African country to another, as when they are shipped outside of the continent. The creation of cell lines falls into these controversies. Both the initial sample collector and their institution may retain interests in or claims of ownership of samples, and collaborators may specify ownership in material transfer agreements for instance. However, the creation and maintenance of cell lines requires significant financial and personnel resources, and these are normally provided by the facility that generated the cell lines. Additionally, in order for the biorepositories to be sustainable, samples and cell lines will likely need to be distributed at a cost. Lastly, some particularly valuable cell lines could be patented – as happened in the case of the HeLa cells, for instance [3]. But generating commercial gain from biological materials is widely recognized to be problematic [9,17-19] – not least because it may be thought to be immoral or illegal to sell and buy body parts and because body parts may be vested with symbolic meaning that ought not be commodified [20,21].

### Perceptions of blood and bodily integrity

An additional challenge in the African context relates to traditional perceptions of blood and the body. It is well-documented that blood carries symbolic value for many Africans [22-26]. Blood has been associated with strength, superstition, exploitative relations, colonialism and witchcraft, amongst others. For this reason, authors highlight the need to carefully explain the purposes of blood collection in the consent process and during research [27,28]. Similarly, other bodily tissues such as organs for transplantation [18], semen for artificial insemination [8], and frozen research samples [13] have also been demonstrated to carry with them aspects of the identity of the donor. An example from Cameroon is the traditional practice that the placenta, or at least a part of it, needs to be buried in the village of origin of the father, even when the family no longer lives there. Whether the same will apply to cell lines, samples collected for routine medical procedures or tissues removed during operations, remains to be investigated in the African context. Another avenue for investigation is whether traditional African perceptions on the afterlife and the spirit world could provide opportunities to discuss ethical issues relating to cell line creation and immortality.

## Consent

The way in which these challenges have traditionally been dealt with is by investing considerable effort in optimising the consent procedure. One of the primary challenges in the creation of cell lines relates to obtaining informed consent for the procedure. Consent for medical research across Africa often remains focused around specific research questions, with samples obtained for use in a particular disease-based project. All of the H3Africa projects, for instance, are disease-based meaning that enrolment and therefore consent is primarily focused on the disease in question. In those cases, sample and data sharing are often of secondary importance. This is significantly different from HapMap and 1000Genomes, where recruitment specifically focused on the creation of cell lines. A decision that needs to be made in the context of H3Africa studies, therefore, is whether consent for the creation of cell lines should be integrated in the main consent form or whether specific consent for cell line creation should be obtained. Greater scrutiny of the HapMap and 1000Genomes experiences may also be required for guidance.

It is clear that against the foregoing discussions, there are many challenges in obtaining informed consent for genomic studies in Africa [28-33]. One pertinent feature of many research contexts in Africa is the complementarity of community and individual values. Community engagement is often a necessary step preceding individual informed consent procedures. In the case of cell line creation, this translates into a requirement to consider how community views can be integrated in sample governance. In light of these concerns, it is therefore unlikely that interactions around informed consent alone can resolve ethical dilemmas around sample sharing and cell line creation. Rather, we need to think creatively to ensure that downstream decisions about the use of cell lines created in H3Africa research, appropriately take into consideration participant interests and are not harmful. For instance, careful development of sample sharing policies, together with clear decisions about appropriate forms of secondary use, are essential accompaniments to informed consent, and have a role to play in protecting participants’ interests.

### Ethics approval and sample access

Cell line creation raises issues that are novel for ethics committees in two ways: first, in terms of the practice itself, and second, in terms of the opportunity if offers for sample re-use. For ethics committees across the continent, the usual practice is to approve the use of samples for specific research projects. Any subsequent uses of those samples need separate approval from that same ethics committee. In the context of H3Africa projects, the proposal is that ethics committees will approve the submission of samples to the biobank, but what remains unresolved is whether those committees will permit another body (such as a sample access committee or an ethics committee associated with the biobank) to make decisions about appropriate secondary uses of samples. It is important to recognise that many participant communities across Africa are considered ‘vulnerable’ to exploitation: poor, with low research literacy and obstructed access to healthcare. In our experience, ethics committees in Africa recognise these contextual issues and take their role in protecting the interests of participants very seriously. Similarly, some ethics committees in Africa have observed that medical research in Africa has yielded little benefit for African patients, institutions and countries, and are becoming increasingly critical about sample sharing and export [15]. Exactly how these concerns will relate to the proposal for wide sample sharing (through cell line creation) remains to be investigated and experienced. What does seem clear, however, is that the large scale creation of cell lines in the context of H3Africa needs to be paired with a clear commitment to building research capacity in Africa. Biobanks can also have an ethics committee that adjudicates over ethical issues such as proposals wishing to create cell lines. In some scenarios once a biobank has been granted ethical clearance as a bona fide biobank then an ethics committee could cede further decisions specific to the highly specialised field of biobanking to the biobanking ethics review committee.

## Summary and recommendations

The creation of cell lines in central biobanks in Africa raises a number of ethical challenges relating to consent, community engagement, ownership and commercialisation and harmonisation of ethics review across the continent. The absence of good regulatory guidance regarding biobanking and its technologies on the continent of Africa raises many additional concerns that also need addressing. Although in this paper, we have presented the various ethical challenges raised by the creation of cell lines as disconnected, they share considerable overlap. For instance, the absence of a good awareness of participants’ understanding of and perspectives on cell line creation raises challenges for ethics review – most evidently in the way ethics committees in African countries seek to prevent exploitation through control over secondary use decisions. Also, the historical exploitation of African people in medical research creates suspicions around blood collection and resistance to sample export. These issues, therefore, need to be understood together rather than in isolation.

There is a considerable gap in knowledge about the perspectives of African research participants on the creation of cell lines for research and therefore an urgent need to conduct empirical studies to investigate these. Such studies could be conducted in the context of current H3Africa initiatives but also, for instance, could involve members of populations for which cell lines are already commercially available. We identify a number of areas that need further work before the large-scale creation of cell lines from human samples could become a reality in Africa. Most importantly what is needed is a comprehensive understanding of the views of stakeholders across the continent on these issues. Empirical research needs to focus on (potential) research participants from diverse populations, in rural and urban settings and from different socio-economic backgrounds. It also needs to encapsulate researchers, medical professionals, ethics committee members and policy makers. Such work needs to span a number of academic disciplines in the social sciences and humanities.

Second, it is necessary to clarify the implications of the current legal frameworks in African countries, or develop these where they are not existent or appropriate. This would involve legal scholars on the continent, as well as policy makers, civil society organisations and others. Furthermore, the role of appropriate governance mechanisms in ensuring that the creation of cell lines is done ethically in a way that builds trust in the research enterprise, needs to be explored. What is clear is that such governance mechanisms must ensure that cell lines are created for and used to promote the wellbeing of patients on the African continent.

Thirdly, attention needs to be given to developing a governing structure that enables scientific research whilst also protecting participant interests. Such a governing structure may require the ongoing involvement of ethics committee members and researchers from across the continent, particularly with regard to access and re-use decisions. In particular, the governance structure should consider how and whether research is likely to benefit African patients and accommodate limitations to informed consent. One suggestion could be that the scientific community revisits the idea of a ‘Hippocratic Oath’ for scientists [34].

In the context of H3Africa, the development of a policy framework to guide the sharing of research samples in biobanks is in its final stages. Also, the Consortium has initiated a process of engagement with members of ethics committees from across the continent to understand the key ethical challenges relating to the sharing of data and samples for genomics research in Africa. From these processes, it is clear that the development of an appropriate consent model to allow for the sharing of samples and the creation of cell lines is a first priority. It is also clear that these activities need to be embedded in robust and thorough community engagement activities, but questions about how community engagement can meaningfully support the creation and redistribution of cell lines require urgent attention. Within H3Africa, some work is taking place to address these issues both through empirical work and through a process of engagement of stakeholders across the continent.

## Endnotes

^a^See http://h3africa.org/about/white-paper (accessed 12 January 2014).

^b^See http://ccr.coriell.org/Sections/BrowseCatalog/Populations.aspx?PgId=4 (accessed 13 January 2013).

^c^See http://www.1000genomes.org/about (accessed 13 January 2014).

## Competing interests

All authors are members of the H3Africa Working Group on Ethics and Regulatory Issues, and are involved in H3Africa projects. They declare that they have no competing financial interests.

## Authors’ contributions

All authors participated in identifying the ethical challenges pertinent to the creation of cell lines. JdV drafted the manuscript, AA, JB, KL, EM, PM, OOMOB and JS reviewed and improved the manuscript. All authors with exception of JdV are listed in alphabetical order. All authors read and approved the final version of this manuscript.

## Authors’ information

Manuscript was prepared by the authors as members of Working Group on Ethics of the H3Africa Consortium.

## Pre-publication history

The pre-publication history for this paper can be accessed here:

http://www.biomedcentral.com/1472-6939/15/60/prepub
